# Prevalence and factors associated with overweight or obesity among 2- to 6-year-old children in Hunan, China: a cross-sectional study

**DOI:** 10.1017/S136898002200012X

**Published:** 2022-12

**Authors:** Na Liu, Huixia Li, Zhanjun Guo, Xin Chen, Peng Cheng, Bian Wang, Guangwen Huang, Minxue Shen, Qian Lin, Jing Wu

**Affiliations:** 1 Department of Endocrinology, Xiang-Ya Hospital, Central South University, Hunan Province 410008, People’s Republic of China; 2 Department of Child Health Care, Hunan Provincial Maternal and Child Health Care Hospital, Changsha, Hunan Province, People’s Republic of China; 3 Department of Social Medicine and Health Management, Xiangya School of Public Health, Central South University, Changsha, Hunan Province, People’s Republic of China; 4 Xiangya School of Public Health, Central South University, Hunan Province, People’s Republic of China; 5 Hunan Engineering Research Center for Obesity and its Metabolic Complications, Changsha, Hunan, People’s Republic of China; 6 National Clinical Research Center for Geriatric Disorders, Xiangya Hospital, Central South University, Changsha, China

**Keywords:** Obesity, Preschool, Childhood, Prevalence, Nutrient, Physical activity

## Abstract

**Objective:**

To compare the prevalence of overweight or obesity (ow/ob) with WHO BMI cut-off points, International Obesity Task Force (IOTF) cut-off points and Chinese BMI criteria and examine its potential factors among preschool children in Hunan Province.

**Design:**

A cross-sectional survey including anthropometric measurements and questionnaires about children’s information, caregivers’ socio-demographic characteristics and maternal characteristics. *χ* ^2^ tests and univariate and multivariate binary logistic regression were performed to evaluate the possible factors of ow/ob.

**Setting:**

Hunan, China, from September to October 2019.

**Participants:**

In total, 7664 children 2 to 6 years of age.

**Results:**

According to Chinese BMI criteria, about 1 in 7–8 children aged 2–6 years had ow/ob in Hunan, China. The overall estimated prevalence of ow/ob among 2- to 6-year-old children was significantly higher when based on the Chinese BMI criteria compared with the WHO BMI cut-off points and IOTF cut-off points. According to Chinese BMI criteria, ow/ob was associated with residing in urban areas, older age, male sex, eating snacking food more frequently, macrosomia delivery, caesarean birth, heavier maternal prepregnancy weight and pre-delivery weight.

**Conclusion:**

The prevalence of ow/ob in preschool children in Hunan Province remains high. More ow/ob children could be screened out according to Chinese BMI cut-offs compared with WHO and IOTF BMI criteria. In the future, targeted intervention studies with matched controls will be needed to assess the long-term effects of intervention measures to provide more information for childhood obesity prevention and treatment.

The prevalence of childhood overweight and obesity has risen in low- and middle-income countries during the past few decades^(1,2)^. The prevalence of obesity is related to genetic and biological factors, as well as socio-environmental factors, including family, school, community and national policy^(3)^. Childhood and adolescent obesity tracks adulthood obesity and has been implicated in many noncommunicable diseases, including type 2 diabetes, hypertension and CVD^(1,4)^. For most noncommunicable diseases caused by obesity, the risks depend partly on the age of onset and on the duration of obesity disease^(1)^. Identifying modifiable risk and protective factors at earlier stages is critical to control obesity epidemics.

BMI is a common indicator of body mass and nutritional status, which is widely used to classify overweight and obesity in adults. BMI as screening for ow/ob in children achieved the continuity in age with adult. The IOTF reference^(5)^ and the WHO BMI criteria^(6,7)^ are the two most common international data sets used to define ow/ob in preschool children. In 2010, China established cut-off points of screening ow/ob for Chinese children and adolescents. The nutrition and health surveys of different diagnostic BMI criteria have reported large disparities in ow/ob rates in Chinese preschool children from different provinces: the overall prevalence of ow/ob ranges from 2·64 % to 18·9 %, as reported in 2006–2017 by IOTF criteria^(8–12)^; the prevalence of obesity among 3- to 6-year ear-old children ranges from 15·9 % to 19·9 % as reported in 2014–2017 by the WHO BMI reference^(13,14)^ and the prevalence of ow/ob among children 3 to 6 years of age ranges from 22·47 % and 26·0 %, as reported in 2017 by Chinese BMI criteria^(10)^. Owing to the heterogeneities in diagnostic criteria and the small sample size^(10,14)^, self-reported height and weight^(10,14)^ and unclear sampling method^(12,14)^, these findings may not reflect the real prevalence of ow/ob.

Children’s nutrition and lifestyles have changed significantly in China. WHO recognises that unhealthy dietary habits, low levels of physical activity (PA), socio-demographics, social and economic development are some of the possible factors for obesity^(1)^. There has been no large-scale survey of the prevalence and contributing factors of obesity in preschool children in Hunan, China, over the past decades. We collected children’s general information, PA and dietary habits, caregivers’ characteristics and socio-demographic characteristics. Dietary patterns consider overall diet rather than individual foods^(15)^; therefore, dietary patterns were derived from a FFQ. Therefore, the aim of the current study was to analyse the occurrence and potential factors of obesity with different criteria in Hunan Province in preschool-age children.

## Methods

### Study design and population

The current study is a cross-sectional survey conducted from September to October 2019 in Hunan Province. Inclusion criteria: (1) child’s age is between 24 and 83 months; and (2) the child should have lived in that location for at least 1 year.

Subjects were selected by multistage stratified cluster sampling. The sample size calculation is given by

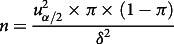




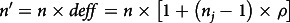

where 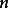 is the sample size of simple randomised sampling, 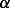 is the inspection level, *α* = 0·05; 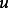 is the boundary value of the standard normal distribution corresponding to *α*, 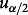 = 1·96; 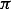 is estimated prevalence of overweight and obesity, π = 0·05 and 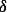 is the allowable error, 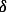 = 0·008. 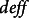 is the design effect, 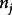 is the average group size, 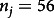 and 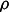 is the correlation coefficient within the group, 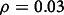 , 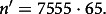 We estimated a rejection rate of 6 %, and the sample size needed 8009 children, considering that some children may refuse to participate or may not complete the questionnaires. The number of clusters is the total number of samples divided by the group size, that is, 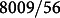 = 143·02. Finally, we sent out 8200 questionnaires from 144 clusters, detailed as follows. Hunan Province is located in the central south of China and is the home of fourteen cities. First, these cities were stratified into low, medium and high economic levels. Then, two cities from each economic level, two urban and two rural districts from each selected city, two streets and two towns from each selected district and three neighbourhood committees and three village committees from each selected town were randomly selected. In total, seventy-two neighbourhood communities and seventy-two village committees were selected. A consecutive sampling survey was used when selecting children from each neighbourhood committee/village committee: the staff of the committee informed the children and caregivers of the local maternal and child health care centre for investigation at a predetermined time in each neighbourhood or village committee. The investigation was completed after the fifty-six children were investigated in order of arrival.

### Questionnaire and definition of variables

We collected each child’s information, including general information, such as gender, birth date, birth weight, mode of delivery, feeding practice for the first six months of life, screen time, time spent outdoors and dietary habits. Birth weights < 2500 g, 2500–3999 g and ≥ 4000 g were considered low birth weight, normal birth weight and macrosomia, respectively^(16)^. The feeding practices for the first six months were divided into exclusive breast-feeding, predominant breast-feeding, exclusive formula feeding and mixed feeding^(17)^: exclusive breast-feeding meant the infant only fed on breast milk, but can be fed with oral rehydration salt, drops, syrups (vitamins, minerals, medicines); predominant breast-feeding meant the infant mainly fed on breast milk, but also took certain liquids (water and water-based drinks, fruit juice), ritual fluids and oral rehydration salt, drops or syrups; mixed feeding meant the infant was fed with both breast milk and non-human milk and formula; formula feeding meant the infant was not fed with breast milk, but only with non-human milk and formula. Dietary habits were evaluated using a thirty-four-item FFQ for the past week based on the Dietary Guidelines for Chinese Residents and the eating habits of Hunan people. Food items were aggregated into eleven food groups based on similar nutrient content (online supplementary material, Supplemental Table 1) as follows: grains, cereals and other starchy staples; tofu and soy products; vegetables; fruits; algae; milk and dairy products; meat and poultry; aquatic; eggs; snacks and beverages, including infant formula (child-specific food items) and removing alcoholic beverages. In the self-reporting questionnaire, the mothers or caregivers were asked whether their children had consumed any of the food items and the frequency of consumption.

Socio-demographic characteristics included residence (urban or rural areas), economic level and parents or caregiver demographic characteristics (i.e. age in years, education and occupation). Maternal characteristics included pregnancy complications (hypertensive disorders of pregnancy, gestational diabetes mellitus, fetal distress and abnormal fetal position), prepregnancy weight, pre-delivery weight and gestational weight gain. Optimal weight gain during pregnancy was determined by the Institute of Medicine guidelines^(18)^: underweight, normal weight, overweight and obese women should gain 12·5–18 kg, 11·5–16 kg, 7–11·5 kg and 5–9 kg during pregnancy. The classification of prepregnancy BMI was based on the Chinese guidelines^(19)^: underweight (BMI < 18·5 kg/m^2^), normal weight (BMI 18·5–23·9 kg/m^2^), overweight (BMI 24·0–27·9 kg/m^2^) and obese (BMI ≥ 28 kg/m^2^).

### Anthropometric measurements

All measurements were taken using the same type of apparatus and followed the same procedures. The children wore only light clothing, without shoes. Height was measured using a telescopic height measuring instrument to the nearest 0·1 cm. Weight was measured using a calibrated beam scale to the nearest 0·05 kg. BMI was calculated as the weight divided by height squared (kg/m^2^).

### Overweight and obesity definition

Under the WHO criteria, BMI for age z score (BAZ) between 2 sd to 3 sd and more than 3 sd was defined as overweight and obesity for children aged 24–60 months^(6)^. BAZ between 1 sd and 2 sd and more than 2 sd was defined as overweight and obesity for children aged 61–83 months^(7)^. The IOTF criteria for overweight and obesity are age and gender specific and based on centile curves that passing through BMI cut-off points for overweight (25 kg/m^2^) and obesity (30 kg/m^2^) at age 18^(5)^. The Chinese criteria define overweight and obesity based on centile curves that passing through BMI cut-off points for overweight (24 kg/m^2^) and obesity (28 kg/m^2^) at age 18 years^(20)^.

### Data collection

Each team consisted of nine data collectors and one supervisor, and everyone received two weeks of training. All study data were double-entered using Epidata3·1 (Jens M. Lauritsen, Michael Bruus, Odense, Denmark) and tested for concordance. For any inconsistent data, the original data were checked to ensure the high accuracy of the inputted data. The original questionnaire was kept in each survey area for verification by the research team.

### Statistical analyses

Statistical analyses were performed using SPSS version 25.0 (IBM) and R, version 4.1.1^(21)^. Frequencies and percentages were calculated for all categorical variables. Pearson’s *χ* ^2^ test was used to compare the prevalence of ow/ob based on different children’s characteristics, socio-demographic characteristics and maternal characteristics. A univariable and multivariable logistic regression model was used to identify significant factors for ow/ob, starting with all variables with *P* < 0·05 in the *χ* ^2^ test. A *P* value of < 0·05 was considered to be statistically significant.

Dietary patterns were identified by factor analysis using the PCA, and varimax rotation was used to rotate the factors to better fit the data. Kaiser–Meyer–Olkin and Bartlett’s test were used to evaluate the reliability and validity of the PCA. The Cronbach’s alpha coefficient were used to evaluate the internal consistency of the dietary questionnaire. Dietary patterns were identified on the basis of the eigenvalue (> 2) and scree plot and named according to the foods with the highest loading on the pattern (factor loading of > |0·30|). We extracted two dietary patterns (see online supplementary material, Supplemental Table 2): the traditional meal pattern and snacking food pattern. The traditional meal pattern was loaded heavily on cereals, soy products, legume, vegetables, fruits, red meat, poultry, seafood, eggs and nuts, while the snacking food pattern was loaded heavily on processed meats, instant noodles, pastries, candy, nuts, fried and puffed food and beverages. Two components explained 14·3 % and 5·7 % of the variance, respectively. Factor scores were divided into four quartiles on the basis of their contribution to each pattern, and an increase from quartile (Q) 1 to Q4 was assumed. Exploratory factor analysis demonstrated the Kaiser–Meyer–Olkin test (*P* = 0·880) and Bartlett’s test ( 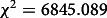 , *P* < 0·001) supported the use of factor analysis. Cronbach’s alpha coefficient (Cronbach’s *α* = 0·826) indicates a good internal consistency of the dietary questionnaire.

The impact of missing data was also considered. Participants with missing data on outcomes – ow/ob (age, height, weight) – were excluded from the analyses. Twenty-two (0·3 %) children had a missing birth weight, 9 (0·1 %) were missing delivery mode, 2 (< 0·1 %) were missing feeding practice for the first six months and 43 (0·6 %) were missing outdoor activity. Father’s and mother’s ages were missing for 155 (2·0 %) and 159 (2·1 %), respectively. A total of 145 (1·9 %) caregivers had a missing education and 170 (2·2 %) were missing occupations. The missing values of hypertensive disorders of pregnancy, gestational diabetes mellitus, fetal distress, disposition, prepregnancy weight and pre-delivery weight were 178 (2·3 %), 170 (2·2 %), 187 (2·4 %), 169 (2·2 %), 18 (0·2 %) and 28 (0·4 %), respectively. On this basis, multiple imputation (mi) was performed using R with the chained equation package^(22)^. The analyses in Tables 1–3 (descriptive analysis, univariable and multivariable logistic regression) were performed with multiple imputation by the chained equations method. In addition, complete data were also used for univariable and multivariable logistic regression (see online supplementary material, Supplemental Table 3).

**Table 1:** Children’s characteristics and univariate analysis of factors associated with ow/ob

Parameters	Total		WHO criteria			IOTF criteria			Chinese criteria		
	*n*	%	*n*	%	*P*-value	*n*	%	*P*-value	*n*	%	*P*-value
Age group (month)					<0·01			<0·01			<0·01
24–35	1625	21·2	36	2·2		46	2·8		131	8·1	
36–47	1830	23·9	62	3·4*		85	4·6*		220	12·0*	
48–59	1999	26·1	65	3·3		126	6·3*,††		270	13·5*	
60–71	1934	25·2	289	14·9*,††,‡‡		217	11·2*,††,‡‡		345	17·8*,††,‡‡	
72–83	276	3·6	55	19·9*,††,‡‡,§§		32	11·6*,††,‡‡		57	20·7*,††,‡‡	
Sex					<0·01			0·02			<0·01
Boys	3920	51·1	335	4·4		285	7·3		655	16·7	
Girls	3744	48·9	172	2·2		221	5·9		368	9·8	
Children’s birth weight (g)					<0·01			<0·01			<0·01
2500–3999	6828	89·1	420	6·2		424	6·2		859	12·6	
< 2500	277	3·6	14	5·1		16	5·8		25	9·0	
≥ 4000	559	7·3	73	13·1†,‡		66	11·8†,‡		139	24·9†,‡	
Mode of delivery					<0·01			<0·01			<0·01
Vaginal	4372	57·0	248	5·7		240	5·5		522	11·9	
Caesarean	3292	43·0	259	7·9		266	8·1		501	15·2	
Feeding practice for the first six months					0·15			0·06			0·42
Exclusive breast-feeding	3835	50·0	232	6·0		224	5·8		493	12·9	
Predominant breast-feeding	1099	14·3	78	7·1		80	7·3		157	14·3	
Mixed feeding	1953	25·5	134	6·9		142	7·3		259	13·3	
Exclusive formula feeding	777	10·1	63	8·1		60	7·7		114	14·7	
Children’s screen time (h/d)					<0·01			<0·01			<0·01
< 1	3095	40·4	152	4·9		162	5·2		356	11·5	
≥ 1	4569	59·6	355	7·8		344	7·5		667	14·6	
Children’s outdoor activity time (h/d)					0·09			0·08			<0·01
< 1	3770	49·2	231	6·1		230	6·1		447	11·9	
≥ 1	3894	50·8	276	7·1		276	7·1		576	14·8	
Children’s traditional meal pattern					0·69			0·58			0·65
Q1	1916	25·0	129	6·7		132	6·9		247	12·9	
Q2	1916	25·0	116	6·1		118	6·2		268	14·0	
Q3	1916	25·0	134	7·0		136	7·1		262	25·6	
Q4	1916	25·0	128	6·7		120	6·3		246	12·8	
Children’s snacking food pattern					<0·01			0·01			<0·01
Q1	1916	25·0	112	5·8		114	5·9		216	11·3	
Q2	1916	25·0	111	5·8		112	5·8		250	13·0	
Q3	1916	25·0	117	6·1		120	6·3		258	13·5||	
Q4	1916	25·0	167	8·7||,¶,§		160	8·4||,¶,§		299	15·6||,¶	

Ow/ob, overweight or obesity; Q, quartile. Data are *n* (%), and *P*-values are *χ* ^2^ test.

*P < 0·05 *v*. 24–35 months.

† *P* < 0·05 *v*. normal birth weight.

‡ *P* < 0·05 *v*. birth weight between 2500 and 3999 g.

§ *P* < 0·05 *v*. Q3.

|| *P* < 0·05 *v*. Q1.

¶ *P* < 0·05 *v*. Q2.

†† *P* < 0·05 *v*. 36–47 months.

‡‡ *P* < 0·05 *v*. 48–59 months.

§§ *P* < 0·05 *v*. 60–71 months.

**Table 2:** Socio-demographic and maternal characteristics and univariate analysis of factors associated with ow/ob

Parameters	Total		WHO criteria			IOTF criteria			Chinese criteria		
	*n*	%	*n*	%	*P*-value	*n*	%	*P*-value	*n*	%	*P*-value
Residence					<0·01			<0·01			<0·01
Urban	3530	46·1	271	7·7		281	8·0		541	15·3	
Rural	4134	53·9	236	5·7		225	5·4		482	11·7	
Economic level					0·61			0·28			0·27
High	2557	33·4	163	6·4		155	6·1		319	12·5	
Moderate	2525	32·9	163	6·5		166	6·6		352	13·9	
Low	2582	33·7	181	7·0		185	7·2		352	13·6	
Father’s age (years)					<0·01			<0·01			<0·01
< 30	1849	24·1	88	4·8		85	4·6		205	11·1	
30–35	3188	41·6	214	6·7†		223	7·0†		452	14·2†	
≥ 35	2627	34·3	205	7·8†		198	7·5†		366	13·9†	
Mother’s age (years)					0·02			0·13			0·47
< 30	1579	20·6	85	5·4		93	5·9		196	12·4	
30–35	3385	44·2	218	6·4		215	6·4		460	13·6	
≥ 35	2700	35·2	204	7·6‡		198	7·3		367	13·6	
Main caregiver					0·15			0·51			0·12
Mother	5092	66·4	330	6·5		331	6·5		687	13·5	
Father	214	2·8	22	10·3		17	7·9		28	13·1	
Grandparents	2314	30·2	151	6·5		153	6·6		297	12·8	
Others	44	0·6	4	9·1		5	11·4		11	25·0	
Caregiver’s education					0·85			0·94			0·96
Primary school or below	1201	15·7	76	6·3		77	6·4		156	13·0	
Middle school	2324	30·3	148	6·4		151	6·5		309	13·3	
High school	2057	26·8	143	7·0		143	7·0		281	13·7	
University or above	2082	27·2	140	6·7		135	6·5		277	13·3	
Caregiver’s occupation					0·49			0·08			0·25
Unemployed	2295	29·9	140	6·1		142	6·2		298	13·0	
Manager	751	9·8	61	8·1		67	8·9		120	16·0	
Technical staff	1246	16·3	83	6·7		79	6·3		171	13·7	
Service works	798	10·4	50	6·3		42	5·3		96	12·0	
Agricultural worker	343	4·5	26	7·6		22	6·4		41	12·0	
Others	2231	29·1	147	6·6		154	6·9		297	13·3	
Pregnancy complications											
HDP					0·38			0·07			0·35
Yes	169	2·2	14	8·3		17	10·1		27	16·0	
No	7495	97·8	493	6·6		489	6·5		996	13·3	
GDM					0·06			<0·01			0·02
Yes	240	3·1	23	9·6		28	11·7		44	18·3	
No	7424	96·9	484	6·5		478	6·4		979	13·2	
Fetal distress					0·19			0·75			0·08
Yes	151	2·0	6	4·0		9	6·0		13	8·6	
No	7513	98·0	501	6·7		497	6·6		1010	13·4	
Abnormal fetal position					0·71			0·52			0·93
Yes	236	3·1	17	7·2		18	7·6		31	13·1	
No	7428	96·9	490	6·6		488	6·6		992	13·3	
Pre-pregnancy weight (kg)					<0·01			<0·01			<0·01
< 50	2735	35·7	123	4·5		120	4·4		256	9·4	
50–55	2399	31·3	153	6·4||		137	5·7||		310	12·9||	
≥ 55	2530	33·0	231	9·1||,¶		249	9·8||,¶		457	18·1||,¶	
Pre-delivery weight (kg)					<0·01			<0·01			<0·01
< 65	3434	44·8	151	4·4		141	4·1		312	9·1	
65–70	1876	24·5	129	6·9*		129	6·9*		257	13·7*	
≥ 70	2354	30·7	227	9·6*,**		236	10·0*,**		454	19·3*,**	
Pre-pregnancy BMI (kg/m^2^)§					<0·01			<0·01			<0·01
< 18·5	737	20·9	41	5·6		40	5·4		80	10·9	
18·5–23·9	2491	70·6	191	7·7		198	7·9		395	15·9††	
≥ 24	302	8·6	39	12·9††,‡‡		43	14·2††,‡‡		66	21·9††,‡‡	
GWG§					<0·01			<0·01			<0·01
Appropriate	1379	39·1	88	6·4		94	6·8		194	14·1	
Inadequate	1028	29·1	71	6·9		69	6·7		121	11·8	
Excessive	1123	31·8	112	10·0‡‡,§§,||||		118	10·5‡‡,§§,||||		226	20·1‡‡,§§,||||	

Ow/ob, overweight or obesity; HDP, hypertensive disorders of pregnancy; GDM, gestational diabetes mellitus; GWG, gestational weight gain. Data are *n* (%), and *P*-values are *χ* ^2^ test.

*P < 0·05 *v*. pre-delivery weight <65 kg.

† *P* < 0·05 *v*. father’s age < 30 years old.

‡ *P* < 0·05 *v*. mother’s age < 30 years old.

§ Mothers in urban areas.

|| *P* < 0·05 *v*. pre-pregnancy weight <50 kg.

¶ *P* < 0·05 *v*. pre-pregnancy weight between 50 and 55 kg.

**P < 0·05 *v*. pre-delivery between 65 and 70 kg.

†† *P* < 0·05 *v*. pre-pregnancy BMI <18·5 kg/m^2^.

‡‡ *P* < 0·05 *v*. pre-pregnancy BMI between 18·5 and 23·9 kg/m^2^.

§§ *P* < 0·05 *v*. appropriate GWG.

|||| *P* < 0·05 *v*. inadequate GWG.

**Table 3:** Univariate and multivariate logistic regression for ow/ob in Chinese children aged 2–6 years

Parameters	WHO criteria				IOTF criteria				Chinese criteria			
	cOR	95 %CI	aOR	95 %CI†	cOR	95 %CI	aOR	95 %CI†	cOR	95 %CI	aOR	95 %CI†
Age group (month)												
24–35	1		1		1		1		1		1	
36–47	1·55	1·02, 2·35*	1·58	1·03, 2·41*	1·67	1·16, 2·41**	1·74	1·20, 2·54**	1·56	1·24, 1·96***	1·59	1·25, 2·01***
48–59	1·48	0·98, 2·24	1·51	0·99, 2·32	2·31	1·64, 3·26***	2·42	1·70, 3·47***	1·78	1·43, 2·22***	1·84	1·46, 2·32***
60–71	7·75	5·45, 11·04***	8·25	5·68, 11·97***	4·34	3·13, 6·01***	4·68	3·31, 6·60***	2·48	2·00, 3·06***	2·56	2·03, 3·22***
72–83	10·99	7·05, 17·11***	11·98	7·50, 19·14***	4·50	2·81, 7·21***	4·81	2·94, 7·88***	2·97	2·11, 4·18***	3·19	2·22, 4·59***
Sex												
Girls	1		1		1		1		1		1	
Boys	1·94	1·61, 2·35***	1·79	1·47, 2·19***	1·25	1·04, 1·50*	1·15	0·95, 1·38	1·84	1·61, 2·11***	1·75	1·52, 2·01***
Children’s birth weight (g)												
2500–3999	1		1		1		1		1		1	
< 2500	0·81	0·47, 1·40	0·89	0·51, 1·59	0·93	0·55, 1·55	0·99	0·59, 1·68	0·69	0·45, 1·05	0·74	0·48, 1·13
≥ 4000	2·29	1·76, 2·98***	1·70	1·27, 2·26***	2·02	1·54, 2·66***	1·52	1·14, 2·03**	2·30	1·87, 2·82***	1·76	1·42, 2·18***
Mode of delivery												
Vaginal	1		1		1		1		1		1	
Caesarean	1·42	1·19, 1·70***	1·25	1·03, 1·51*	1·51	1·26, 1·81***	1·31	1·09, 1·59**	1·32	1·16, 1·51***	1·19	1·03, 1·36*
Children’s screen time (h/d)												
< 1	1		1		1		1		1		1	
≥ 1	1·63	1·34, 1·98***	1·03	0·84, 1·28	1·47	1·22, 1·79***	1·04	0·84, 1·27	1·32	1·15, 1·51***	1·02	0·88, 1·18
Children’s outdoor activity time (h/d)												
< 1	1		1		1		1		1		1	
≥ 1	1·17	0·98, 1·40	1·12	0·93, 1·36	1·17	0·98, 1·41	1·15	0·95, 1·39	1·29	1·13, 1·47***	1·25	1·09, 1·43**
Children’s snacking food pattern												
Q1	1		1		1		1		1		1	
Q2	0·99	0·76, 1·30	0·92	0·69, 1·22	0·98	0·75, 1·28	0·90	0·69, 1·19	1·18	0·97, 1·43	1·15	0·94, 1·40
Q3	1·05	0·80, 1·37	0·92	0·69, 1·22	1·06	0·81, 1·38	0·93	0·71, 1·22	1·23	1·01, 1·49*	1·14	0·93, 1·39
Q4	1·54	1·20, 1·97***	1·35	1·04, 1·76*	1·44	1·12, 1·85***	1·23	0·95, 1·59	1·46	1·21, 1·76***	1·32	1·09, 1·61**
Residence												
Rural	1		1		1		1		1		1	
Urban	1·37	1·15, 1·65***	1·36	1·09, 1·70**	1·50	1·25, 1·80***	1·47	1·18, 1·82***	1·37	1·20, 1·57***	1·37	1·17, 1·60***
Father’s age (years)												
< 30	1		1		1		1		1		1	
30–35	1·44	1·12, 1·86**	1·04	0·77, 1·41	1·56	1·21, 2·02***	1·26	0·94, 1·69	1·33	1·11, 1·58***	1·11	0·91, 1·37
≥ 35	1·69	1·31, 2·19***	0·95	0·65, 1·39	1·69	1·30, 2·20***	1·07	0·73, 1·55	1·30	1·08, 1·56**	0·91	0·69, 1·19
Mother’s age (years)												
< 30	1		1		1		1		1		1	
30–35	1·21	0·94, 1·57	0·97	0·72, 1·31	1·08	0·84, 1·39	0·88	0·66, 1·18	1·11	0·93, 1·33	1·00	0·81, 1·23
≥ 35	1·44	1·10, 1·87	1·03	0·71, 1·49	1·26	0·98, 1·63	0·94	0·66, 1·35	1·11	0·92, 1·34	0·98	0·75, 1·27
GDM												
No	1		1		1		1		1		1	
Yes	1·52	0·98, 2·36	1·42	0·88, 2·29	1·92	1·28, 2·88***	1·71	1·12, 2·61*	1·48	1·06, 2·06*	1·29	0·91, 1·82
Pre-pregnancy weight (kg)												
< 50	1		1		1		1		1		1	
50–55	1·45	1·13, 1·85***	1·24	0·95, 1·62	1·32	1·03, 1·70*	1·13	0·86, 1·48	1·44	1·21, 1·71***	1·24	1·03, 1·50*
≥ 55	2·13	1·70, 2·67***	1·46	1·09, 1·95*	2·38	1·90, 2·98***	1·61	1·21, 2·15**	2·14	1·81, 2·52***	1·46	1·18, 1·80***
Pre-delivery weight (kg)												
< 65	1		1		1		1		1		1	
65–70	1·61	1·26, 2·05***	1·34	1·02, 1·77*	1·73	1·35, 2·21***	1·38	1·05, 1·82*	1·59	1·33, 1·89***	1·36	1·12, 1·65***
≥ 70	2·32	1·88, 2·87***	1·88	1·42, 2·49***	2·60	2·10, 3·23***	1·79	1·36, 2·37***	2·39	2·05, 2·79***	1·77	1·45, 2·17***

Results of univariate and multivariate logistic regression analyses in various significant factors. Analyses with multiple imputation data. cOR, crude OR. aOR, adjusted OR. GDM, gestational diabetes mellitus; Q, quartile.

*P < 0·05, **P < 0·01, ***P < 0·005.

† Adjusts for all variables with *P* < 0·05 in the *χ* ^2^ test.

## Results

### Prevalence of overweight and obesity among 2- to 6-year-old children

In this survey, we distributed a total of 8200 questionnaires. A total of 463 children’s data could not be evaluated because of refusal to participate or not completing the questionnaire, and thus we received 7737 (94·4 %) questionnaires. After excluding ineligible child age (≥ 7 years or < 2 years) (*n* 17) and incomplete anthropometric measurements (*n* 56), the present analysis involved a total of 7664 children.

**Fig. 1:**
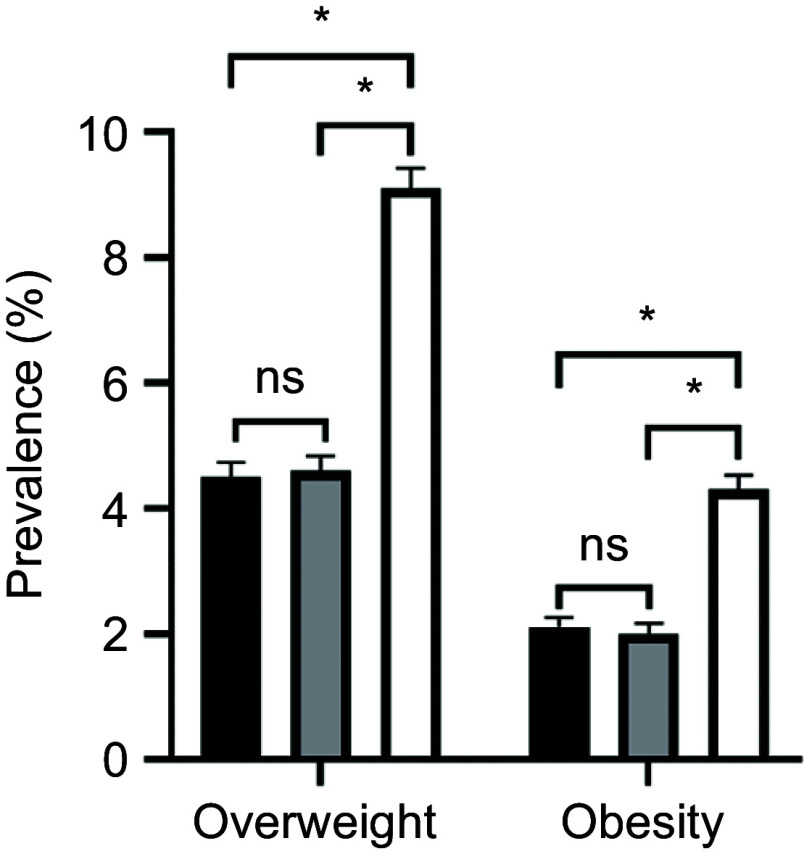
Prevalence of overweight and obesity among 2- to 6-year-old children in Hunan Province using the WHO, IOTF and Chinese BMI criteria. *P < 0·05. 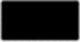, WHO criteria; 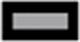, IOTF criteria; 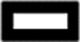, Chinese criteria

The estimated prevalence of overweight and obesity was significantly higher when based on the Chinese criteria (overweight: 9·1 %, 95 % CI: 8·8 %, 9·4 %; obesity: 4·3 %, 95 % CI: 4·1 %, 4·5 %) compared with the WHO (overweight: 4·5 %, 95 % CI: 4·3 %, 4·7 %; obesity: 2·1 %, 95 % CI: 1·9 %, 2·3 %) and IOTF (overweight: 4·6 %, 95 % CI: 4·4 %, 4·8 %; obesity: 2·0 %, 95 % CI: 1·8 %, 2·1 %) cut-off points (Fig. 1). The number of ow/ob children screened using the WHO (*n* 507) and IOTF (*n* 506) criteria is about half that of those screened by Chinese criteria (*n* 1023).

### Population characteristics and univariate analysis of factors associated with ow/ob

Tables 1 and 2 show the study population characteristics and univariate analysis of factors associated with ow/ob. The 72- to 83-month-old group had the fewest children, mainly because children over the age of 6 have basically entered primary school. Half of the children were reported to have been exclusive breastfed. Nearly half (42·9 %) of the children were delivered by caesarean delivery (Table 1). Among the three categories of diagnostic standards, the estimated prevalence of ow/ob children under 60 months old was the lowest based on WHO criteria, and the estimated prevalence of ow/ob children aged 60–83 months was the lowest based on IOTF criteria. Ow/ob children were significantly older, had heavier birth weights, were more likely to live in urban areas and were delivered by caesarean delivery (Tables 1 and 2).

### Univariate and multivariate binary logistic analysis for potential ow/ob factors among children aged 2–6 years in Hunan, China

Model covariates were age group (24–35 months, 36–47 months, 48–59 months, 60–71 months, 72–83 months); sex (girls, boys); children’s birth weight (2500–3999 g, < 2500 g, ≥ 4000 g); mode of delivery (vaginal, caesarean); children’s screen time (< 1 h/d, ≥ 1 h/d), time spent outdoors (< 1 h/d, ≥ 1 h/d), children’s snacking food pattern (Q1, Q2, Q3, Q4); residence (rural, urban); father’s age (< 30 years, 30–35 years, ≥ 35 years); mother’s age (< 30 years, 30–35 years, ≥ 35 years); gestational diabetes mellitus (no, yes); prepregnancy weight (< 50 kg, 50–55 kg, ≥ 55 kg) and pre-delivery weight (< 65 kg, 65–70 kg, ≥ 70 kg).

Under logistic regression, crude OR and adjusted OR showed little difference between the two approaches (multiple imputation analysis (Table 3) and complete-case analysis (see online supplementary material, Supplemental Table 3)). Univariate and multivariate binary logistic regression analyses (Table 3) demonstrated the associations between significant factors and ow/ob using different references. Our results suggest that, according to Chinese BMI criteria, residence, sex, age, child birth weight, mode of delivery, time spent outdoors, maternal prepregnancy weight and pre-delivery weight and snacking food pattern were statistically significant variables. Children in the highest quartile (Q4) of the snacking food pattern had greater odds of ow/ob than those in the lowest quartile (Q1).

## Discussion

BMI is the most commonly used simple measure of weight status, while there is not any consistent standard about childhood obesity. Two international criteria are used to define ow/ob for preschool children based on BMI: The IOTF reference is based on children under 25 years old from six national cross-sectional studies to define childhood overweight and obesity^(5)^ while the WHO published a growth standard for children aged 0–5^(6)^ and 5–19 years^(7)^, which is based on samples of children who represent optimal growth. However, weight status in children and adolescents is complicated by racial and regional differences^(23)^. In 2010, the Chinese BMI cut-offs for screening ow/ob were established based on the BMI growth reference values^(20)^, which was obtained from two large national surveys of 69 760 children. It has been gradually used in domestic epidemiological studies in recently years^(24–26)^. We then employed the above three criteria (WHO, IOTF and Chinese criteria) to screen overweight and obesity. Our results show that the detection rate of ow/ob in preschool children is the highest by the Chinese criteria. The number of ow/ob children screened by Chinese criteria is about twice that screened by WHO or IOTF criteria. Similar trends have been reported, as demonstrated by a study from Asia, including China, Lebanon and Malaysia, showing that approximately one-third and half of obese Asian children aged 8–10 years were not screened using WHO and IOTF standards, respectively^(27)^. This suggests that the application of the Chinese criteria may be more appropriate to screen for obesity-related risks in Chinese children.

In our study, the prevalence of ow/ob among preschool children in Hunan was lower than those in other provinces of China. Compared with previous studies using WHO BMI criteria, the prevalence of ow/ob we surveyed (7·8 %, age 3–6 years; 3·3 %, age 3–4 years; 15·6 %, age 5–6 years) was significantly lower than that in Tianjin in 2014 (7·4 %, age 3–4 years; 35·0 %, age 5–6 years)^(13)^ and Beijing in 2017 (9·2 %, age 3–4 years; 22·1 %, age 5–6 years)^(14)^. The prevalence of obesity we surveyed was significantly lower than that of the China Health and Nutrition Survey in 2011 (12·6 %, age 3–6 years)^(9)^, Liaoning Province in 2008–2009 (6·08 %, age 2–7 years)^(11)^ and Beijing in 2017 (8·46 %, age 3–6 years)^(10)^ according to the IOTF criteria. Surprisingly, the obesity prevalence of preschool children living in the urban areas we reported (2·4 %, aged 2–6 years) was slightly lower than the National Epidemiological Survey on Simple Obesity in Childhood in 2006 (2·64 %, age 3–7 years)^(8)^ by the IOTF criteria, representing the prevalence of childhood obesity in a Chinese city region. The prevalence of ow/ob we surveyed (14·8 %, age 3–6 years) was significantly lower than that in Changsha in 2017 (26·0 %, age 3–6 years)^(28)^ and Beijing in 2017 (23·0 %, age 3–6 years)^(10)^ by Chinese BMI criteria. Beijing, Tianjin, Liaoning and Hunan per capita GDP ranked in the top 1, 3, 13 and 16 in China,^(29)^ respectively, and Changsha’s GDP per capita 2018 ranked among the top of Hunan Province^(30)^. A national epidemiological survey on obesity in children under seven years of age in nine cities of China in 2016 found that the prevalence of obesity in the central region (4·4 %) was lower than that in the north (4·7 %) but higher than that in the south (3·6 %)^(31)^. The cities mentioned above (Beijing, Tianjin, Liaoning) are located in northern China, and Hunan is located in central China. We infer that the prevalence of childhood obesity in Hunan Province remains at a relatively low level compared with other urban areas in China, probably partly related to the lower macrosomia proportion (466/3176 *v*. 557/7664)^(8)^, lower caesarean delivery proportion (537/1123 *v*. 1123/7664)^(14)^, relatively lower level of economic development and geographic differences.

The prevalence of childhood obesity was higher in girls than in boys among European children aged 2–7 years^(32)^. However, our study shows that boys have a higher prevalence of obesity, corroborating other findings in China^(9–11,13)^. Gender disparity in obesity could be explained by numerous factors^(33,34)^: Chinese girls are more likely to keep a slender shape, and boys spend more time playing computer games. In addition, Chinese families always think of the larger, more muscular ideal male body shape, and they are less likely to encourage sons to lose weight due to traditional gender preferences for boys. Therefore, Chinese families should enhance their feeding knowledge and pay more attention to children’s weight status, especially boys.

In several developed areas, there was a reversal in urban–rural disparity, with the prevalence of overweight and obesity in rural children surpassing than that of their urban peer^(35)^. In our work, urban children were more likely to be obese than rural children. One possible explanation is that the rapid urbanisation and industrial development in China has led to changes in human activity and diet structure^(36)^: individuals living in urban areas have less occupational and daily PA that relies on motorised and labour-saving transport or appliances, and they consume greater amounts of high-energy-density foods than their rural counterparts; this excess energy is unlikely to be compensated through leisure physical activities.

Kindergarten Work Regulations^(37)^ pointed out that time spent outdoors for children (including outdoor sports activities) shall not be < 2 h a day. Our survey shows that nearly half of children spend less than 1 h outdoors and children who spend more time outdoors have a higher prevalence of ow/ob. Most researchers believe that increased PA could prevent weight gain and alleviate the obesity epidemic^(38,39)^. More time outdoors does not connote more exercise, although outdoor space could provide the environment for PA^(40,41)^. The relationship between time spent outdoors and obesity needs to be further clarified by evaluating the time, intensity and form of PA in the future.

Dietary patterns consider overall diet rather than individual foods; therefore, they may better define to the public how to interpret or translate into diets and provide guidance for nutrition intervention and education^(15)^. We evaluated the effects of eating frequency of traditional meal and snack food on body weight and concluded that higher frequency of eating snack food was associated with ow/ob. Our study shows that the snacking food pattern was heavily loaded by the consumption of cakes, pastries, candy, fried food and beverages, which are rich in fats and sugar and low in fibre. A previous study showed that energy-dense snack food (e.g. sweets, cakes and pastries) consumption was associated with higher energy intake, poor nutrients and obesity^(42)^. Beverage consumption cannot reduce the intake of other foods because of its poor satiety compared with solid food^(43)^. The sugar content of most foods and beverages was considerably underestimated by parents^(44)^, providing easily accessible and practicable knowledge about sugar content may help parents choose healthier foods for their children. Caregivers and kindergarten teacher should encourage children to develop good eating habits and strictly limit snacks for children.

Previous research observed that the association between caesarean section and higher risk of offspring obesity attenuated but remained statistically significant after adjusting for maternal pre-pregnancy BMI^(45)^, which may be related to the different gastrointestinal microbiota established at birth for different delivery mode^(46)^. Maternal obesity may increase the risk of caesarean delivery and macrosomia^(47)^. Our study shows that children with caesarean birth and heavier maternal weights are more likely to suffer from obesity, although the heights of the mothers in rural areas were not collected. Next, the height of the mother can be collected to analyse the relationship between maternal obesity, caesarean section and obesity in offspring. Thus, we consider that it is wise that proper nutrition during pregnancy and prevention of excessive birth weight should not be overlooked in preventing childhood obesity.

Evidence shows that poverty and low socio-economic status may contribute to an epidemic of childhood and adolescent obesity^(48)^. In accordance with the previous findings reported, caregivers with lower education may contribute to the development of child ow/ob because they usually have less health knowledge, lower income, insecure food, etc.^(49–51)^. In the current study, we did not observe consistent associations between ow/ob and different economic levels, caregivers’ education or occupation. Similarly, a study evaluated the impacts of different socio-economic indicators (such as parental education and occupation and socioeconomic status) on abdominal obesity and found that obesity is associated with socio-economic indicators in higher income countries, but this association was not observed in a lower-middle-income country, a possible reason for this discrepancy may be related to the different economic levels. Accordingly, a holistic approach that considers socio-economic background and family income and education will be helpful for better understanding the prevalence of overweight and obesity in children.

We know that the current study has several limitations. First, a cross-sectional study cannot verify causality. Second, the data collection on maternal weight was based on caregiver recall, which unavoidably led to recall bias, and the heights of the mother in rural areas were not collected, which could not be used to analyse the relationship between maternal BMI and offspring obesity. However, our analysis indicates that mothers who had heavier prepregnancy BMI or gestational weight gain were positively related to children’s ow/ob in urban areas, which can partially explain the relationship between them. Finally, the use of a single frequency of food consumption for the past week was not adequate to account for the daily energy consumption, and the two dietary patterns explained only 20·0 % of the total variance; thus, it is possible that other dietary patterns existed in the current study population. Nevertheless, this large-scale epidemiological survey involved 7664 children of age 2–6 years in Hunan Province. We employed three different criteria to define ow/ob simultaneously and identified several individual and socio-demographic factors, which is conducive to the early identification and intervention of overweight and obesity.

In conclusion, this large-scale epidemiological survey shows that the prevalence of ow/ob in preschool children in Hunan Province remains high. More ow/ob children could be screened out using Chinese BMI cut-offs compared with WHO and IOTF BMI criteria. Tailored interventions that encourage mothers to maintain healthy weight, choose vaginal delivery, increase health knowledge, create a healthy food environment to promote the development of healthy lifestyles for children, especially pay more attention to boys, may be important in reducing the risk of child ow/ob. In the future, targeted intervention studies with matched controls will be needed to assess the long-term effects of intervention measures to provide more information for childhood obesity prevention and treatment.

## Supplementary material

For supplementary material accompanying this paper visit https://doi.org/10.1017/S136898002200012X. click here to view supplementary material
