# Supplementary material for: Prevalence and factors associated with overweight or obesity among 2- to 6-year-old children in Hunan, China: a cross-sectional study
Source: Public Health Nutr. 2022 Jan 17;25(12):3487–98. doi: 10.1017/S136898002200012X (PMC9991611; doi:10.1017/S136898002200012X)
Supplement: Supplementary file 1 [file S136898002200012Xsup001.docx]

Supplementary table 1. Foods included in FFQ

| **Food group** | **Food items** | **Examples** |
| --- | --- | --- |
| Grains | Cereals | rice, rice congee |
|  | Rice noodles, noodles |  |
|  | Pasta | steamed bun, bread roll, pancake |
|  | Corn and coarse grain | millet, corn, sorghum rice, buckwheat |
|  | Starchy roots and tubers | potato, sweet potato, purple sweet potato, taro |
| Tofu and soy products | Tofu |  |
|  | Soy milk |  |
|  | Other legume products | yuba, tofu sheet, silken tofu, dry tofu |
| Vegetables | Vegetables rich in vitamin A, dark green leafy | green pepper, broccoli, spinach, snow peas, Chinese chives, leaf lettuce, ect. |
|  | Vegetables rich in vitamin A, orange-yellow leafy | tomato, carrot, pumpkin, ect. |
|  | Other Vegetables | cucumber, eggplant, celery stalk, white radish, lotus root, sprout, lettuce, onion, asparagus, wax gourd, kidney bean, towel gourd, ect. |
| Fruit | Fruits rich in vitamin A | orange, mango, pawpaw, Hami melon |
|  | Other fruits | apple, pear, grapefruit, peach, strawberry, grape, banana, Chinese date, kiwi fruit, pitaya, watermelon, pomegranate, persimmon, plum, apricot, pineapple, ect. |
| Algae | Fungus and Seaweed | mushroom, agaric, tremella, nori, kelp, flammulina velutipes, ect. |
| Milk and dairy products | Infant formula |  |
|  | Ordinary milk powder |  |
|  | Fresh milk |  |
|  | Yogurt |  |
|  | Solid or semi-solid dairy products | cheese |
| Meat | Red meat | Pork, beef, lamb, etc.rou |
|  | Poultry | chicken, duck, geese, etc |
|  | Processed meats | sausage, lunchmeat |
|  | Animal liver, animal blood products |  |
| Aquatic | Fish |  |
|  | Shrimp and crab |  |
|  | Other seafood | Jellyfish, shellfish, |
| Eggs | Eggs | eggs, duck eggs, quail eggs, pidan, etc. |
| Snack | Instant noodles |  |
|  | Pastries | bread, biscuits |
|  | Candy | chocolate, candy, honey, jam, jelly |
|  | Nuts | melon seeds, peanuts, walnuts, pistachios, hazelnuts, cashews, etc. |
|  | Fried and puffed food | chips, crisps, etc. |
|  | Solid drinks | ice cream, popsicle, etc |
| Beverage | Carbonated drinks | Cola, Sprite, Fanta and other soft drinks |
|  | Fruit and vegetable juice drinks |  |
|  | Milk beverages | lactobacillus drink, milky drinks |
|  | Vegetable protein drink | apricot kernel juice, walnut drink, peanut sauce |
|  | Sugar-sweetened tea drinks |  |
|  | Sugar-free drinks |  |

Supplementary Table 2. Factor-loading matrix for the two dietary patterns and their food group

| **Dietary patterns** | **Food group** | **Factor-loading^*^** | |
| --- | --- | --- | --- |
| Traditional meal pattern | Cereals | | 0.324 |
|  | Corn and coarse grain | | 0.402 |
|  | Starchy roots and tubers | | 0.438 |
|  | Tofu | | 0.402 |
|  | Soy milk | | 0.355 |
|  | Other legume products | | 0.413 |
|  | Vegetables rich in vitamin A, dark green leafy | | 0.593 |
|  | Vegetables rich in vitamin A, orange-yellow leafy | | 0.629 |
|  | Other Vegetables | | 0.603 |
|  | Fruits rich in vitamin A | | 0.573 |
|  | Other fruits | | 0.535 |
|  | Fungus, and Seaweed | | 0.522 |
|  | Red meat | | 0.439 |
|  | Poultry | | 0.478 |
|  | Fish | | 0.384 |
|  | Shrimp and crab | | 0.416 |
|  | Other seafood | | 0.317 |
|  | Eggs | | 0.332 |
|  | Nuts | | 0.34 |
| Snacking food pattern | Processed meats | | 0.454 |
|  | Instant noodles | | 0.407 |
|  | Pastries | | 0.344 |
|  | Candy | | 0.478 |
|  | Nuts | | 0.355 |
|  | Fried and puffed food | | 0.576 |
|  | Solid drinks | | 0.488 |
|  | Carbonated drinks | | 0.513 |
|  | Fruit and vegetable juice drinks | | 0.423 |
|  | Milk beverages | | 0.452 |
|  | Vegetable protein drinks | | 0.433 |
|  | Sugar-sweetened tea drinks | | 0.51 |
|  | Sugar-free drinks | | 0.312 |

**^*^**Factor loading of >|0.30| was included to represent the food strongly associated with the identified factor; absolute values <0.30 are excluded from the table for simplicity.

Supplementary Table 3. Univariate and multivariate logistic regression for ow/ob in Chinese children aged 2-6 years

| **Parameters** | **WHO criteria** | | **IOTF criteria** | | **Chinese criteria** | |
| --- | --- | --- | --- | --- | --- | --- |
|  | cOR (95%CI) | aOR (95%CI)^#^ | cOR (95%CI) | aOR (95%CI) ^#^ | cOR (95%CI) | aOR (95%CI) ^#^ |
| Age group (month) |  |  |  |  |  |  |
| 24-35 | 1 | 1 | 1 | 1 | 1 |  |
| 36-47 | 1.55(1.02-2.37) ^*^ | 1.56(1.01-2.41) ^*^ | 1.64(1.13-2.38) ^**^ | 1.70(1.56-2.49) ^**^ | 1.53(1.21-1.94) ^***^ | 1.56(1.22-1.99) ^***^ |
| 48-59 | 1.45(0.95-2.12) | 1.45(0.94-2.24) | 2.30(1.62-3.26) | 2.39(1.66-3.44) ^***^ | 1.76(1.40-2.20) ^***^ | 1.81(1.42-2.29) ^***^ |
| 60-71 | 7.55(5.27-10.81) ^***^ | 7.82(5.36-11.43) ^***^ | 4.26(3.06-5.92) ^***^ | 4.52(3.18-6.41) ^***^ | 2.41(1.94-3.00) ^***^ | 2.46(1.94-3.12) ^***^ |
| 72-83 | 10.64(6.76-16.76) ^***^ | 11.29(6.98-18.26) ^***^ | 4.39(2.71-7.12) ^***^ | 5.58(2.77-7.60) ^***^ | 2.92(2.05-4.15) ^***^ | 3.10(2.13-4.51) ^***^ |
| Sex |  |  |  |  |  |  |
| Girls | 1 | 1 | 1 | 1 | 1 | 1 |
| Boys | 2.00(1.65-2.44) ^***^ | 1.87(1.52-2.29) ^***^ | 1.31(1.09-1.58) ^**^ | 1.21(1.00-1.47) | 1.90(1.65-2.19) ^***^ | 1.82(1.57-2.10) ^***^ |
| Children’s birth weight (g) |  |  |  |  |  |  |
| 2500-3999 | 1 | 1 | 1 | 1 | 1 | 1 |
| <2500 | 0.67(0.36-1.23) | 0.71(0.38-1.34) | 0.78(0.44-1.37) | 0.81(0.46-1.45) | 0.58(0.37-0.92) ^*^ | 0.61(0.38-0.98) ^*^ |
| ≥4000 | 2.15(1.63-2.83) ^***^ | 1.54(1.14-2.08) ^**^ | 1.96(1.48-2.61) ^***^ | 1.44(1.07-1.94) ^*^ | 2.32(1.88-2.86) ^***^ | 1.75(1.40-2.18) ^***^ |
| Mode of delivery |  |  |  |  |  |  |
| Vaginal | 1 | 1 | 1 | 1 | 1 | 1 |
| Caesarean | 1.43(1.19-1.73) ^***^ | 1.25(1.03-1.53) ^*^ | 1.52(1.26-1.83) ^***^ | 1.31(1.08-1.59) ^**^ | 1.33(1.16-1.53) ^***^ | 1.18 (1.03-1.37) ^*^ |
| Children’s screen time (hours/day) |  |  |  |  |  |  |
| <1 | 1 | 1 | 1 | 1 | 1 | 1 |
| ≥1 | 1.70(1.38-2.08) ^***^ | 1.10(0.88-1.37) | 1.50(1.23-1.83) ^***^ | 1.06(0.86-1.32) | 1.36(1.18-1.56) ^***^ | 1.06(0.91-1.24) |
| Children’s outdoor activity (hours/day) |  |  |  |  |  |  |
| <1 | 1 | 1 | 1 | 1 | 1 | 1 |
| ≥1 | 1.17(0.97-1.41) | 1.12(0.92-1.36) | 1.18(0.98-1.42) | 1.16(0.96-1.40) | 1.29(1.12-1.48) ^***^ | 1.24(1.08-1.43) ^***^ |
| Children’s snacking food pattern |  |  |  |  |  |  |
| Q1 | 1 | 1 | 1 | 1 | 1 | 1 |
| Q2 | 0.98(0.74-1.29) | 0.90(0.67-1.21) | 0.99(0.75-1.30) | 0.91(0.68-1.20) | 1.18(0.97-1.44) | 1.14(0.93-1.40) |
| Q3 | 1.04(0.79-1.37) | 0.93(0.69-1.23) | 1.07(0.81-1.40) | 0.94(0.71-1.24) | 1.21(0.99-1.48) | 1.13(0.92-1.39) |
| Q4 | 1.55(1.20-1.99) ^***^ | 1.38(1.05-1.81) ^*^ | 1.46(1.13-1.89) ^***^ | 1.27(0.97-1.65) | 1.44(1.19-1.74) ^***^ | 1.32(1.08-1.61) ^**^ |
| Residence |  |  |  |  |  |  |
| Rural | 1 | 1 | 1 | 1 | 1 | 1 |
| Urban | 1.33(1.10-1.60) ^***^ | 1.34(1.06-1.69) ^*^ | 1.47(1.22-1.77) ^***^ | 1.46(1.17-1.83) ^**^ | 1.36(1.19-1.56) ^***^ | 1.39(1.17-1.63) ^***^ |
| Father’s age (years) |  |  |  |  |  |  |
| <30 | 1 | 1 | 1 | 1 | 1 | 1 |
| 30-35 | 1.42(1.09-1.84) ^**^ | 1.00(0.74-1.37) | 1.53(1.18-1.99) ^***^ | 1.22 (0.90-1.64) | 1.29(1.08-1.55) ^**^ | 1.06(0.86-1.32) |
| ≥35 | 1.67(1.28-2.17) ^***^ | 0.91(0.61-1.35) | 1.68(1.28-2.19) ^***^ | 1.03(0.70-1.52) | 1.29(1.07-1.55) ^**^ | 0.87(0.66-1.15) |
| Mother’s age (years) |  |  |  |  |  |  |
| <30 | 1 | 1 | 1 | 1 | 1 | 1 |
| 30-35 | 1.19(0.91-1.55) | 0.97(0.71-1.33) | 1.08(0.83-1.39) | 0.89(0.66-1.19) | 1.07(0.89-1.29) | 0.98(0.79-1.22) |
| ≥35 | 1.44(1.10-1.89) ^**^ | 1.07(0.73-1.58) | 1.28(0.99-1.66) | 0.98(0.67-1.41) | 1.11(0.92-1.34) | 1.00(0.76-1.32) |
| GDM |  |  |  |  |  |  |
| No | 1 | 1 | 1 | 1 | 1 | 1 |
| Yes | 1.53(0.97-2.39) | 1.42(0.88-2.32) | 1.92(1.27-2.90) ^***^ | 1.68(1.09-2.59) ^*^ | 1.54(1.09-2.15) ^*^ | 1.32(0.92-1.88) |
| Prepregnancy weight (kg) |  |  |  |  |  |  |
| <50 | 1 | 1 | 1 | 1 | 1 | 1 |
| 50-55 | 1.48(1.15-1.91) ^***^ | 1.27(0.97-1.68) | 1.34(1.04-1.74) ^**^ | 1.16(0.88-1.53) | 1.52(1.27-1.82) ^***^ | 1.32(1.08-1.61) ^**^ |
| ≥55 | 2.15(1.71-2.72) ^***^ | 1.48(1.10-2.00) ^*^ | 2.39(1.90-3.14) ^***^ | 1.63(1.22-2.18) ^***^ | 2.21(1.86-2.62) ^***^ | 1.50(1.21-1.86) ^***^ |
| Predelivery weight (kg) |  |  |  |  |  |  |
| <65 | 1 | 1 | 1 | 1 | 1 | 1 |
| 65-70 | 1.59(1.24-2.04) ^***^ | 1.33(1.00-1.76) | 1.71(1.33-2.20) ^***^ | 1.36(1.03-1.81) ^*^ | 1.63(1.36-1.95) ^***^ | 1.37(1.12-1.69) ^***^ |
| ≥70 | 2.35(1.89-2.92) ^***^ | 1.90(1.43-2.54) ^***^ | 2.60(2.09-3.25) ^***^ | 1.79(1.35-2.38) ^***^ | 2.46(2.09-2.89) ^***^ | 1.80(1.47-2.22) ^***^ |

Note: Results of univariate and multivariate logistic regression analyses in various significant factors. Analyses with complete data. cOR, crude odds ratio. aOR, adjusted odds ratio. CI, confidence interval. GDM: gestational diabetes mellitus. Q: quartile. ^⁎^p < 0.05. ^**^p < 0.01. ^⁎⁎⁎^ p < 0.005. ^#^adjusts for all variables with p <0.05 in the chi-squared test.
